# Supplementary material for: Psychological State and Exam Performance among Paramedics’ Students in Geneva during the COVID-19 Pandemic: A Mixed Methods Study
Source: Int J Environ Res Public Health. 2023 Feb 20;20(4):3736. doi: 10.3390/ijerph20043736 (PMC9959811; doi:10.3390/ijerph20043736)
Supplement: Supplementary file 1 [file ijerph-20-03736-s001.zip › File S3. Original French version of the guide used to conduct the semi-structured interviews, and its English translation.pdf]

Table S1. Original French version of the guide used to do the semi-structured interviews, and its English translation

Original French version	English translated version
Nom	Name
Âge	Age
Volée	Promotion
1. Comment avez-vous vécu le confinement ?	
a. Êtes-vous senti stressé ?	a. Did you feel stressed?
i. Oui -> comment avez-vous ressenti ce stress (physiquement, psychologiquement) ?	i. Yes -> how did you feel about this stress (physically, psychologically)?
ii. Avez-vous eu une perte de plaisir ?	ii. Have you experienced a loss of pleasure?
iii. Avez-vous eu perte d'intérêt ?	iii. Did you lose interest?
iv. Ce stress a-t-il eu un impact dans votre vie privée ?	iv. Has this stress had an impact on your private life?
v. Quel impact cela a eu sur votre manière d'apprendre ?	v. What impact has this had on the way you learn?
vi. Qu'est-ce que vous avez fait pour être moins stressé ?	vi. What have you done to become less stressed?
b. Avez-vous eu peur ?	b. Were you afraid?
i. Oui -> Quelle était votre peur ?	i. Yes-> What was your fear?
ii. Comment avez-vous ressenti votre peur (physiquement, psychologiquement) ?	ii. How did you feel about your fear (physically, psychologically)?
iii. Quel impact cela a eu sur votre manière d'apprendre ?	iii. What impact has this had on the way you learn?
iv. Avez-vous fait quelque chose pour la diminuer ?	iv. Have you done anything to reduce it?
c. Avez-vous eu de la joie ?	c. Did you have joy?
i. Oui -> Comment s'est-elle manifestée ?	i. Yes -> How did it manifest itself?
d. Vous êtes-vous senti anxieux ?	d. Did you feel anxious?
i. Oui -> Comment s'est manifestée votre anxiété ?	i. Yes -> How did your anxiety manifest itself?
ii. Cette anxiété a-t-elle eu un impact dans votre vie privée ?	ii. Has this anxiety had an impact on your private life?
iii. Quel impact cela a eu sur votre manière d'apprendre ?	iii. What impact has this had on the way you learn?
iv. Avez-vous fait quelque chose pour la diminuer ?	iv. Have you done anything to reduce it?
e. Vous êtes-vous senti triste ?	e. Did you feel sad?
i. Oui -> Comment s'est-elle manifestée ?	i. Yes -> How did it manifest itself?
ii. Cette tristesse a-t-elle eu un impact dans votre vie privée ?	ii. Has this sadness had an impact on your private life?
iii. Quel impact cela a eu sur votre manière d'apprendre ?	iii. What impact has this had on the way you learn?
iv. Avez-vous fait quelque chose pour la diminuer ?	iv. Have you done anything to reduce it?
f. Vous êtes-vous senti en colère ?	f. Did you feel angry?
i. Oui -> Comment s'est-elle manifestée ?	i. Yes -> How did it manifest itself?
ii. Cette colère a-t-elle eu un impact dans votre vie privée ?	ii. Has this anger had an impact on your private life?
iii. Quel impact cela a eu sur votre manière d'apprendre ?	iii. What impact has this had on the way you learn?
iv. Avez-vous fait quelque chose pour la diminuer ?	iv. Have you done anything to reduce it?
2. Êtes-vous senti seul (dimension soutien social) ?	2. Did you feel lonely (social support dimension)?
a. Oui ->Vous viviez seul ?	a. Yes ->Did you live alone?
b. Avez -vous un réseau d'amis important ?	b. Do you have a large network of friends?
c. Sur combien d'amis fidèles pouvez-vous compter (au sens de l'ami·e que vous pourriez réveiller au milieu de la nuit) ?	c. How many loyal friends can you count on (in the sense of a friend you could wake up in the middle of the night)?
d. Votre famille se compose-t-elle de personnes ressources pouvant vous aider à apprendre ?	d. Does your family have any resource people who can help you learn?
i. Si oui combien ?	i. If yes, how many?
ii. Frère / sœur ?	ii. Brother / sister?
iii. Père / mère ?	iii. Father / mother?

iv. Autres (cousins) ?	iv. Other (cousins)?
v. Quel est leur niveau de formation ?	v. What is their level of education?
3. Comment se passaient les cours à distance (théoriques) ?	3. How did you experience the (theoretical) distance learning courses?
4. Qu'est-ce qui vous manquaient le plus en ayant les cours en visioconférence ?	4. What did you miss the most about having the videoconference courses?
5. Aviez-vous un environnement propice à l'apprentissage (au domicile) ?	5. Did you have a supportive learning environment (at home)?
i. Pourquoi ?	i. Why?
ii. Vous viviez seul ? chez vos parents ? en colocation ?	ii. Did you live alone? with your parents? in a shared flat?
6. Les cours en visioconférence ont-ils eu un impact sur votre motivation ?	6. Did the videoconference courses have an impact on your motivation?
i. Pourquoi ?	i. Why?
7. Avez-vous eu la possibilité d'étudier/ apprendre /travailler avec d'autres étudiants, enseignants ou autres ?	7. Have you had the opportunity to study/learn/work with other students, teachers, or others?
i. Avez-vous des personnes ressources/ privilégiées dans la classe pour vous aider en cas de besoins ?	i. Do you have any resource/priority people in the class to help you if you need it?
8. Avez-vous gardé contact avec vos collègues de classe durant les cours à distance ?	8. Did you keep in touch with your classmates during the distance learning courses?
i. Si oui, par quel moyen ?	i. If yes, by what means?
a. Numérique, présentiel ?	a. Digital, face-to-face?
b. Cela été un soutien émotionnel pour vous ?	b. Was it an emotional support for you?
c. Cela été un soutien d'apprentissage ?	c. Was it a learning support?
d. Avez-vous pu le trouver vers quelqu'un d'autre ?	d. Were you able to find it to someone else?
9. Formation pratique (stage et pratique simulée à l'école)	9. Practical training (internship and simulated practice at school)
i. Avez-vous été affecté (confinement) sur le nombre d'heures de formation pratique ?	i. Have you been affected (lockdown) on the number of hours of practical training?
a. Avez-vous l'impression que cela à eu un impact sur le développement de vos compétences pratiques (gestes techniques etc) ?	a. Do you feel that this has had an impact on the development of your practical skills (technical skills etc)?
b. Oui-> Sentiez-vous moins à l'aise en pratiquant des gestes techniques (voie veineuse, planchage…) ou bien sur l'évaluation primaire, anamnèse ?	b. Yes-> Do you feel less comfortable performing technical procedures (IV-line, immobilization...) or primary assessment, history taking?
c. Avez-vous eu des stages annulés ? Si oui combien de jours /semaines ?	c. Have you had any internships cancelled? If yes, how many days/weeks?
d. Avez-vous travaillé à la place dans un service d'ambulance ?	d. Did you work instead in an ambulance service?
i. Oui -> Pensez-vous que cela a été bénéfique pour votre apprentissage pratique ?	i. Yes -> Do you think it was beneficial for your practical learning?
e. Considérez-vous que l'annulation de stages ont eu un impact sur le développement de vos compétences professionnelles ? Si oui de quelle manière ? Comment avez-vous compensé ce manque ?	e. Do you consider that the cancellation of internships had an impact on the development of your professional skills? If so, in what way? How did you compensate for this?
f. Etiez-vous moins engagé dans la formation ? Avez-vous eu l'envie d'arrêter la formation ?	f. Were you less involved in the curriculum? Did you feel like stopping the curriculum?
g. Avez-vous senti une différence une fois sur un nouveau lieu de stage ?	g. Did you feel any difference once you were in a new internship location?
h. (jeunes diplômés) Considérez-vous que le confinement, le COVID-19 a eu un impact sur la valeur de votre diplôme ?	h. (recent graduates) Do you consider that the lockdown, the COVID-19 has had an impact on the value of your degree?
i. En gros ton diplôme il est tout pourri ?	i. Basically your degree is all rotten?
10. Pendant cette période de pandémie, beaucoup de techniques ont été utilisées, selon vous y'a-t-il des techniques à garder qui ont favorisé votre apprentissage ?	10. During this period of pandemic, many techniques were used, are there any techniques that you think you should keep that helped you learn?
