# Supplementary material for: Psychological State and Exam Performance among Paramedics’ Students in Geneva during the COVID-19 Pandemic: A Mixed Methods Study
Source: Int J Environ Res Public Health. 2023 Feb 20;20(4):3736. doi: 10.3390/ijerph20043736 (PMC9959811; doi:10.3390/ijerph20043736)
Supplement: Supplementary file 1 [file ijerph-20-03736-s001.zip › File S6. Semi-structured interviews retranscription.pdf]

Annexes-Guide d’entretien

Etudiant 1

Âge : 24 ans
Volée : 19-22

1. **Comment avez-vous vécu le confinement ?** “Mal,c’est le premier mot qui me vient. Très dure à suivre les cours, à se concentrer sur ce qu’on fait. Toujours de la distraction à la maison, un classique quoi ... Pour dire le reste, même l’isolement de pas voir les gens, c’est différent. Il y a l’impression d’avoir moins de contact. tu parles sur des groupes mais c’est pas aussi bien que d'habitude peu l'essentiel.”
2. **Êtes-vous senti stressé ?** “Oui”
    a. **Oui->Comment avez-vous ressenti ce stress (physiquement, psychologiquement) ?** “Sur le renferment un peu, tu t'isoles, genre tu as plus envie de voir les gens. À la maison, tu restes dans ta chambre..des choses comme ça.”
    b. **Avez-vous eu une perte de plaisir ?** “Pas sur ,non je pense pas, pas pour ce que j’aime faire d’habitude”
    c. **Avez-vous eu perte d'intérêt ?** “Oui, possible”
    d. **Ce stress a-t-il eu un impact dans votre vie privée ?** “Oui du coup, l’isolement, ça devient très tendu et ouais c’est comme ça.”
    e. **Quel impact cela a eu sur votre manière d’apprendre ?** “Rien à changer du tout.”
    f. **Qu'est-ce que vous avez fait pour être moins stressé ?** ”Bah ouais en fait, tu t'enfuies dans quelque chose d'autre, tu fais des activités où tu t’évades. Le sport, ou des séries, des trucs comme ça. C’est pas vraiment de réduire le stress mais de l’oublier”
3. **Avez-vous eu peur ?** “Non”
4. **Avez-vous eu de la joie ?** “Moins que d’habitude je pense”
    a. **Oui -> Comment s’est-elle manifestée ?** “Alors ça.... j’en sais rien du tout”
5. **Êtes-vous senti anxieux ?** “Oui”
    a. **Oui->Comment s’est manifestée votre anxiété ?** “De nouveau la même chose, tu te renferme, mal au ventre, c’est les deux choses principales.”
    b. **Cette anxiété a-t-elle eu un impact dans votre vie privée?** “Oui, sur les relations avec les gens, tu t'éloignes un peu de tout le monde même inconsciemment.”
    c. **Quel impact cela a eu sur votre manière d’apprendre ?** “Tu arrives plus à te concentrer,tu es en permanence anxieux et puis tu arrives plus à te plonger ni dans les cours ni dans rien, car c’est pas une période toute rose et ça donne pas envie de t’enfermer dans des révisions, de bosser tout le temps.”**Question en plus : Vous étiez anxieux de quoi ?** “De pas réussir dû à ses cours à distances et pis ses stages faussées par le covid.”

   d. **Avez-vous fait quelque chose pour la diminuer ?** “Je voyais une psy à ce moment-là mais je la voyais déjà avant, donc ça aide je pense. Mais c’est pas pour cette période là que je la voyais”
6. **Êtes-vous senti triste?** “Non”
7. **Êtes-vous en colère?** “Non”
8. **Êtes-vous senti seul (dimension soutien social) ?** “Oui, comme je disais tu t'éloignes, tu vois beaucoup moins les gens. Tout le monde essaye de gérer le confinement comme il peut et puis ça éloigne un peu tout le monde.”
   a. **Oui ->Vous viviez seul ?** “Non”
   b. **Avez -vous un réseau d'amis important ?** “Oui”
   c. **Sur combien d'amis fidèles pouvez-vous compter (au sens de l’ami.e que vous pourriez réveiller au milieu de la nuit) ?** 6
   d. **Votre famille se compose-t-elle de personnes ressources pouvant vous aider à apprendre ?** “Oui”
      i. **Si oui combien ?** “2”
      ii. **Frère / sœur ?**
      iii. **Père / mère ?** “Oui les deux”
      iv. **Autres (cousins) ?** “non”
      v. **Quel est leur niveau de formation ?** “Diplôme HES”
9. **Comment se passaient les cours à distance (théoriques)?** “Il s'passait! Non, c’était, enfin pour moi, j’ai des grosses difficultés à suivre quand c’est en visio. Je me suis vu plusieurs fois me déconcentrer après 10 min et puis plus rien écouter pour le reste du cours. Du coup, je devais reprendre les cours après car je n’avais pas entendu la moitié.”
10. **Qu’est-ce qui vous manquaient le plus en ayant les cours en visioconférence ?** “Les interactions, je pense, parce qu’elles sont très difficiles en visio. On te pose des questions, c’est vachement plus dur de prendre la parole, et du coup ça fausse vachement l’activité du cours. Et puis, avoir quelqu'un en présentiel ça te stimule….autres choses que de voir un écran, une voix qui parle par-dessus.”
11. **Aviez-vous un environnement propice à l’apprentissage (au domicile) ?** “Si non ou oui , pourquoi ? en terme de calme oui, en terme de distraction autour vue que c’était dans ma chambre pas du tout . Il aurait fallu que je sorte tout ce qu’il y a dans ma chambre.”
   a. **Vous viviez seul ? Chez vos parents ? En colocation ?** “chez les parents”
12. **Les cours en visioconférence ont-ils eu un impact sur votre motivation?** “Je pense pas mais ça demande deux fois plus de boulot et tu dois refaire tout les cours car c’est très difficile de suivre mais sinon je crois pas.”
13. **Avez-vous eu la possibilité d’étudier/ apprendre /travailler avec d’autres étudiants, enseignants ou autres ?** “Oui”
14. **Avez-vous des personnes ressources/ privilégiées dans la classe pour vous aider en cas de besoins ?** “Oui”
15. **Avez-vous gardé contact avec vos collègues de classe durant les cours à distance?** “Oui”
   a. **Si oui, par quel moyen ?** "Whatsapp, et puis même en présentiel de temps en temps.”
   b. Numérique, présentiel ?
   c. **Cela été un soutien émotionnel pour vous ?** “Oui je pense, tout le monde est dans la même galère, ça aide.”

d. **Cela été un soutien d’apprentissage ? “**Aussi oui”
e. **Avez-vous pu le trouver vers quelqu’un d'autre ?** ”Non pas besoin”

Formation pratique (stage et pratique simulée à l’école)

16. **Avez-vous été affecté (confinement) sur le nombre d’heures de formation pratique ?** “Oui “
17. **Avez-vous l’impression que cela à eu un impact sur le développement de vos compétences pratiques (gestes techniques etc)?** “Non”
18. **Avez-vous eu des stages annulés ? Si oui combien de jours /semaines? “**Non”
19. **Avez-vous travaillé à la place dans un service ?** “Non”
20. **Etiez-vous moins engagé dans la formation ?** “Ca pu arriver mais pas en permanence, mais la motivation avec le covid ça a changer genre les inter plus trop diversifier pendant deux mois de stage c’est compliqué quoi”
21. **Avez-vous eu l’envie d’arrêter la formation ?** “non”
22. **Avez-vous senti une différence une fois sur un nouveau lieu de stage ? Comment ?** “Oui, bah l’encadrement différent, les référents sont malades, le suivi est très difficile, et vu qu’ils doivent gérer une pandémie et en plus gérer des étudiants bah tu ressens que tu es pas mis en premier plan dans l’encadrement”
23. **Après cette période de pandémie, beaucoup de techniques ont été utilisées, selon vous y'a-t-il des techniques à garder qui ont favorisé votre apprentissage ?**
“Les e-learning, moi j’arrive mieux à apprendre quand on dirige les révisions, et puis le reste ça a pas fondamentalement changer.”

Etudiante 2

Âge: 24 ans
Volée : 19-22

1. **Comment avez-vous vécu le confinement de manière générale? “**Pour moi c’était clairement attendu, la fermeture des écoles on savait que tout allait fermé après. Pour cela qu’on a été boire une dernière quinch le jeudi soir. Ca faisait très peur. Ca faisait très catastrophe, fin du monde. J’ai le souvenir d’aller faire la queue à la migros et qu’il y avait ces annonces dans les hauts-parleurs : “en raison de la situation pandémique, voyez porter votre masque” ça faisait pour moi les film science fiction avec les marchés vides, ça faisait peur dans l’ambiance et il y avait pleins incertitudes, on ne savait pas ce que c’était ? Mais j’ai le souvenir d Véronique qui était venue nous voir juste avant le confinement qui nous disait “ le monde va changer les gars “, parce qu’elle faisait que des courses avec les tyveks non-stop et qu’elle arrêtait pas de bosser et cela faisait très peur dans ce sens-là. J’étais aussi inquiète sur le fait que je faisais beaucoup de chose, je bougeais beaucoup, je sortais beaucoup, et le fait d’être enfermé même si c’était pas vraiment dans la loi qu'on devait être enfermé mais je m'inquiétais beaucoup de m’ennuyer, de tourner en rond et tout et au final ça m’est assez convenu de passer du temps chez moi. Je crois que ça faisait des années que je n'avais pas vraiment apprécié être chez moi à chiller chez moi, en pyjama, en training. Et au final, j’ai réalisé que j’aimais bien tout ça et que maintenant je suis beaucoup plus casière qu’avant. Je l’ai aussi assez bien vécu parce que Julien était là, j‘étais pas juste toute seul avec ma mère, on jouait à la switch avec Julien. Et lui,il était encore en stage et y avait aussi ce côté où il allait travailler et le soir quand il revenait il avait des trucs à nous raconter du monde extérieur autre que j’ai été faire la queue à la migros et du coup ça m'a aidé.”
2. **Êtes-vous senti stressé ?** “Surtout au début, de nouveau ça faisait très peur, on savait pas trop à quoi s’attendre…au début j’étais très stressé mais une fois que ça c’est un peu tassé, un peu plus posé et un peu plus de directives et que ça touchait plus les personnes à risque, âgées etc… Là, j’étais moins stressé et je l’ai plus mieux vécu et plus par rapport à la formation de ce qu’on ratait à l’école mais moins dans le contexte pandémique dans le cadre de l’école que sur la pandémie.”
    a. **Oui->Comment avez-vous ressenti ce stress (physiquement, psychologiquement) “**Je pense qu’il y avait beaucoup plus de psychologique et physiquement ça allait mais il y avait aussi l’impact sur le faite que je bougeais moins, j’ai pris pas mal de poids et ça, ça me stressait beaucoup aussi enfin d'être entrain de larver à la maison et pas pouvoir faire du sport, de sortir. Plus psychologique mais aussi des causes physiques.”
    b. **Avez-vous eu une perte de plaisir ?** “Oui, j’ai pu apprécier de nouvelle chose: rester en pyjama, jouer à des jeux vidéo et faire des puzzles mais j’ai perdu du plaisir à sortir voir des gens, boire des verres, sortir après les cours , d’être aussi dans cette ambiance de classe.”
    c. **Avez-vous eu perte d'intérêt ? “**Non, je ne crois pas”

d. **Ce stress a-t-il eu un impact dans votre vie privée ?** “Des fois j’étais un peu plus tendu, ce qui crée un peu plus de tension mais rien de dramatique et pas tous les jours.”
e. **Quel impact cela a eu sur votre manière d’apprendre ?** “Pas directement en lien avec le stress.”
f. **Qu'est-ce que vous avez fait pour être moins stressé ?** “Des puzzle, des activités qui me changeaient les idées, de m’occuper. J'ai commencé la broderie, le tricot, j’ai acheté la switch. Je faisais les puzzles, je faisais beaucoup d’activités bricolage, casanière qui m’occupais l’esprit, ce qui m’évitait de tourner en rond.”

3. **Avez-vous eu peur ?** “Au début surtout, au début du contexte et après j’ai eu peur pour les études.”
    a. **oui-> Quelle était votre peur ?** “Le contexte et les études.”
    b. **Comment avez-vous ressenti votre peur (physiquement, psychologiquement)**? “C’était psychologique, pas tellement psychosomatique.”
    c. **Quel impact cela a eu sur votre manière d’apprendre ? “**Non je crois pas”
    d. **Avez-vous fait quelque chose pour la diminuer ? “**Comme avant, de nouveau d’essayer dans discuter et faire des activités pour passer le temps, pour m'occuper de penser à autres choses.”
4. **Avez-vous eu de la joie ? “**Oui de nouveau, je pense que j’ai apprécié de nouvelle chose que je faisais pas avant : d’être dans mon lit à lire des livres, j’ai redécouvert cette joie là.”
    a. **oui -> Comment s’est-elle manifestée ?** “De nouveau, sur l’humeur de manière générale, y a des choses que j'appréciais moins avant que j’ai plus appris à apprécier maintenant, encore aujourd’hui.”
5. **Êtes-vous senti anxieux ? “**Oui début par le contexte et après par les études, très anxieuse par rapport aux études, une fois qu'on était vraiment dedans.”
    a. **Oui->Comment s’est manifestée votre anxiété ? “**De nouveau plus psychologique que psychosomatique. J’étais très inquiète qu’on devienne incompétant, et qu’on soit pas…. qu’on sort de l’école que soit moins bon que les autres..”
    b. **Cette anxiété a-t-elle eu un impact dans votre vie privée ? “**Non”
    c. **Quel impact cela a eu sur votre manière d’apprendre ? “**Je pense qu’il y avait un peu plus de stress,parce que du fait des visio c'était super difficile de se concentrer mais en même temps j’avais la culpabilité de plus s’impliquer que d’habitude plus pour les rares cours qu’on avait, qui étaient données par visio.”
    d. **Avez-vous fait quelque chose pour la diminuer ?** “Comme avant”
6. **Êtes-vous senti triste?** “Non, pas spécialement triste”
7. **Êtes-vous en colère? “**Je crois qu’il y a eu un moment où j’étais très en colère, c’est quand on nous a annoncé par mail qu'il avait annoncé au service qui fermait les stage mais que nous on avait pas eu cette infos mais que nos stages n’étaient pas annulés.. Les infos ne sont pas passé, ça m'était très en colère.”
    a. **Oui -> Comment s’est-elle manifestée ? “**J’ai ragé avec Ben.”
    b. **Cette colère a-t-elle eu un impact dans votre vie privée ? “**Non”

c. **Quel impact cela a eu sur votre manière d'apprendre ? "**Perdre un peu foi en l'école quand il ya des trucs comme ça pas que tu crois plus du tout au système mais… je crois que c'est la première fois que j'ai commencé à remettre en question l'école..tu sais tu es tellement content de rentrer dans cette école et pis je me suis dit c'est le premier trucs qui merde. T'es un peu moins congruent avec l'école."
d. **Avez-vous fait quelque chose pour la diminuer ? "**J'ai bu une bière et rager avec Ben"

8. **Êtes-vous senti seul (dimension soutien social) ?** "Oui"
   a. **oui ->Vous viviez seul ? "**non"
   b. **Avez -vous un réseau d'amis important ? "**oui"
   c. **Sur combien d'amis fidèles pouvez-vous compter (au sens de l'ami.e que vous pourriez réveiller au milieu de la nuit)** ? "3-4"
   d. **Dans quel sens, vous avez senti la solitude ? "**Dans le sens que j'avais beaucoup d'activités sociales ou j'allais volontiers boire une bière après les cours avant, voir du monde et cela me manquait , les rassemblement en groupe ça me manquaient vraiment."
   e. **Votre famille se compose-t-elle de personnes ressources pouvant vous aider à apprendre ?** "Oui"
      i. **Oui combien ? "**1"
      ii. **Frère / sœur ?** "Non"
      iii. **Père / mère ?** "Mère"
      iv. **Quel est leur niveau de formation ?** "Prof à l'université"
9. **Comment se passaient les cours à distance (théoriques) ? "**Très mal, j'ai déjà du mal à me concentrer en présentiel où souvent je dois faire autre chose en même temps parce que j'ai besoin que ça aille vite et qui se passe des choses. Souvent j'ai besoin de faire une petite activité secondaire pour suivre. Et en visio encore plus, d'être toute seule dans ma chambre face à mon écran, j'arrivais pas à me concentrer du tout."
10. **Qu'est-ce qui vous manquaient le plus en ayant les cours en visioconférence ?** "Le cadre je pense. Le cadre d'être dans une classe, là pour apprendre. T'es à l'école sur un bureau de classe,tu as ton prof qui est là, tu es dans le cadre de l'école pour apprendre et quand t'es chez toi en pyjama dans ta chambre c'est pas la même chose. Et aussi le contact avec les gens, de voir les gens au pause, de pouvoir discuter ça aussi ça manquait."
11. **Aviez-vous un environnement propice à l'apprentissage (au domicile)**
    a. **Si non ou oui , pourquoi ?** "Oui et non , il me faudrait une pièce rien que pour ça, il faudrait pas que ce soit dans ma chambre ou le salon ou la cuisine. Mais oui, car y' a trop de stimulis externe et ni d'enfant qui crie, ou des gens qui me dérange pendant que mes cours . J'ai trop de distractions chez moi aussi."
    b. **Vous viviez seul ? Chez vos parents ? En colocation ?** "Chez mes parent"
12. **Les cours en visioconférence ont-ils eu un impact sur votre motivation ?**
    a. **Si non ou oui , pourquoi ?** "Oui, car vu que j'avais plus de mal à me concentrer et que le format ne me convenait pas, bah ça me motivait moins."

13. **Avez-vous eu la possibilité d'étudier/ apprendre /travailler avec d'autres étudiants, enseignants ou autres ? "**Je crois pas, oui mais pas exploiter, peu de cours où il fallait réviser en soi."
14. **Avez-vous des personnes ressources/ privilégiées dans la classe pour vous aider en cas de besoins ?** "Pierre pour les connaissances et Ben pour la proximité et le fait de voir quelqu'un."
15. **Avez-vous gardé contact avec vos collègues de classe durant les cours à distance?**
    a. **Si oui, par quel moyen ?** "Oui certains"
    b. **Numérique, présentiel ?** "Réseaux sociaux et présentiel"
    c. **Cela été un soutien émotionnel pour vous ?** "Oui"
    d. **Cela été un soutien d'apprentissage ?** "Non pas spécialement"
    e. **Avez-vous pu le trouver vers quelqu'un d'autre, le soutien d'apprentissage ?** "Oui vers Julien"

Formation pratique (stage et pratique simulée à l'école)

16. **Avez-vous été affecté (confinement) sur le nombre d'heures de formation pratique ?** "Oui, j'ai été très frustré par le fait qu'on dise d'apprendre sur le tas et que quand on est arrivé en deuxième année au cours bloc trauma, on s'est fait engueuler car on était pas compétent alors que c'était les heures qu'on avait raté."
17. **Avez-vous l'impression que cela à eu un impact sur le développement de vos compétences pratiques (gestes techniques etc)? "**Oui je pense."
    a. **Oui-> Vous sentez-vous moins à l'aise en pratiquant des gestes techniques (voie veineuse, planchage…) ou bien sur l'évaluation primaire, anamnèse ? "**Plus tôt geste pratique trauma."
18. **Avez-vous eu des stages annulés ? Si oui combien de jours /semaines? "**Non"
19. **Avez-vous travaillé à la place dans un service ?** "Non"
20. **Etiez-vous moins engagé dans la formation ?** "Oui par manque de motivation."
21. **Avez-vous eu l'envie d'arrêter la formation ?** "Non"
22. **Avez-vous senti une différence une fois sur un nouveau lieu de stage ?** "Non, je crois pas mais peut être plus chouchoutés que les autres, je sais pas si c'était une bonne ou mauvaise chose."
23. **Après cette période de pandémie, beaucoup de techniques ont été utilisées, selon vous y'a-t-il des techniques à garder qui ont favorisé votre apprentissage ?** "Seule les choses bien c'est les cours enregistrés qu'on peut réécouter."

Etudiante 3

Âge : 26 ans
Volée : 18-21

1. **Comment avez-vous vécu le confinement ?** “Au début, c'était une période très stressante parce que ma maman est immunosupprimée. Et du fait d’être en pandémie avec ce virus, c’était quelque chose de très stressant pour elle, pour qu’elle l’attrape et que j’étais potentiellement un vecteur. Après j’ai dû déménager et vivre chez ma meilleure amie.Et du coup c’était très chouette,parce que du coup je ne me suis pas sentie seule. Elle vivait avec son copain et on a eu des très chouettes moments qu’on a pu passer ensemble, où j’ai pu être aussi tranquille par rapport à ma maman. Dès le début du confinement, j’ai très rapidement commencé à travailler en P3 donc je ne me suis pas sentie du tout isolée à aucun moment. J’avais vraiment l’impression de pouvoir continuer à être active, d’avoir la chance de pouvoir travailler en ambulance parce que nos stages ont tous été annulés à cette période. J’ai pu prendre le temps de travailler sur mon analyse de pratique professionnelle."
2. **Êtes-vous senti stressé ? “**Au début oui, par rapport au fait que j’habitais à la maison, puis après j’essayais quand même de faire attention par rapport à mes colocataires vu que j’avais commencé en ambulance et qu’on avait vraiment beaucoup de cas covid, aux alentours de 6-7 par jour même en P3, du coup c’était toujours de faire attention à ça qui était stressant. J’essayais vraiment de maintenir les restrictions au niveau sanitaire au maximum pour protéger mes colocs.”
    a. **Oui -> Comment avez-vous ressenti ce stress (physiquement, psychologiquement) ?** “Alors psychologique et que pour un très court moment, vraiment début de la pandémie, au début du travail et au début du confinement”
    b. **Avez-vous eu une perte de plaisir ?** “Non”
    c. **Avez-vous eu perte d'intérêt ? “**Non plus “
    d. **Ce stress a-t-il eu un impact dans votre vie privée ? “** Non, enfin je pense que l’impact était chez tout le monde dans le sens plus personne ne se voyait donc pas plus de stress par rapport à mon fonctionnement.”
    e. **Quel impact cela a eu sur votre manière d’apprendre ?** “Vue qu’on s’est retrouvé très vite autonome sur ce qu’on devait faire, dans le sens on était laissé à nous-même, fallait…on était très libre en termes d’organisation, donc j’ai pu autant travailler en ambulance que pour l’école mais ce qui était difficile c’était de s’organiser avec cette période de libre. On ne savait pas combien de temps ça allait durer, il fallait en même temps anticiper pour la suite et en même temps fallait se mettre des échéances seuls, parce que sinon c’était trop facile de lâcher prise par rapport à l’école.”
    f. **Qu'est-ce que vous avez fait pour être moins stressé ?** “Je pense qu’on en a beaucoup discuté au début avec ma mère et après coloc de comment est-ce qu’on allait faire pour l’organisation de la vie en commun par rapport au fait que je travaillais avec des patients covid en ambulance. Et puis après j’ai fait un planning pour le reste du

confinement pour les échéances que je mettais pour mon analyse de pratique professionnelle pour être sûr d'être dans les temps. Et puis à côté j'essayais de faire du sport à la maison et garder un équilibre entre une activité physique et le travail que je devais fournir pour l'école,"

3. **Avez-vous eu peur ?** "Alors pas pour moi mais plus pour mes proches et surtout ma maman. Et il y a un moment, où je me suis demandé des conditions sanitaires où ça allait aller, jusqu'à où on allait être confronté à devoir trier des patients. C'était un peu la chose, c'était pas vraiment une peur, mais d'anticipation que ça devienne pire. Et c'était compliqué pour moi de se dire de commencer la vie professionnel et de devoir trier des patients et faire le moins de chose possible et être seul avec des patients sans mes collègues et de faire faux"
    a. **Oui->Comment avez-vous ressenti votre peur (physiquement, psychologiquement)?** "Psychologiquement "
    b. **Quel impact cela a eu sur votre manière d'apprendre ?** "Non pas du tout"
    c. **Avez-vous fait quelque chose pour la diminuer ?** "Comment dire, c'était pas… je ne pense pas que peur soit le bon sentiment que je décrirais car finalement, c'était la latence avec la situation sanitaire, je faisais au jour le jour. Je peux pas dire que j'ai fait quelque chose ou que je pouvais faire quelque chose, voir comment ça allait se développer"
4. **Avez-vous eu de la joie ?"**oui"
    a. **Oui -> Comment s'est-elle manifestée ?"** Par beaucoup de moment chouette avec ma meilleure pote et son copaine "
5. **Êtes-vous senti anxieux ?** "Non"
6. **Êtes-vous senti triste?** "Non pas du tout "
7. **Êtes-vous en colère?** "Non"
8. **Êtes-vous senti seul (dimension soutien social) ?** "Non heureusement grâce au fait d'avoir déménagé avec ma meilleure pote, je pense ça m'a vraiment aidé à maintenir équilibre et puis le fait de travailler, t'es dans un contexte où tu as du monde, tu es quand même en présence d'autre gens, donc j'avais pas l'impression seul ce qui était très agréable."
    a. **Oui ->Vous viviez seul ?** "En coloc"
    b. **Avez -vous un réseau d'amis important ?** "Oui"
    c. **Sur combien d'amis fidèles pouvez-vous compter (au sens de l'ami.e que vous pourriez réveiller au milieu de la nuit) ?** "Une dizaine"
    d. **Votre famille se compose-t-elle de personnes ressources pouvant vous aider à apprendre ?** "Non"
9. **Comment se passaient les cours à distance (théoriques) ?** "Ca dépend du format de cours,les formats de cours que j'ai le plus apprécié c'était ceux où les enseignants et enseignantes ont enregistré leur cours, ce qui était hyper pratique, on pouvait mettre pause revenir et si on avait pas compris quelques choses on pouvait revenir dessus et ça, c'est vraiment la méthode, qui moi m'a plus plus. Les cours en visioconférence avec tout le monde c'était hyper compliqué avec des problèmes de connexion de réseau, des problèmes d'ordinateur, des problèmes de sons. C'était toute une logistique, plus compliqué avec beaucoup moins d'interactions entre les gens, soit tout le

monde se coupait la parole soit personne ne parlait. Et il n'y avait pas ce temps entre on se lève et le moment où on fait le trajet pour aller à l'école et du coup on est moins réveillé. Pis tout d'un coup on se retrouve à faire de l'école à la maison….ouais les mondes ne sont plus séparés et il y a une autre dynamique à trouver "

10. **Qu'est-ce qui vous manquaient le plus en ayant les cours en visioconférence ?** "L'interaction et puis on se sentait beaucoup moins légitime de poser des questions. On ne comprenait pas forcément tout,bah au niveau des problèmes techniques, on ne voyait pas forcément tous les documents qui étaient projetés. De part l'interaction, tu as aussi beaucoup moins de concentration pour moi, c'était beaucoup plus dur de rester accrocher à un cours et de rester attentif."
11. **Aviez-vous un environnement propice à l'apprentissage (au domicile) ?**
    a. **Si non ou oui , pourquoi ?**" Pas chez moi, mais heureusement que j'ai pu déménager et être chez ma coloc, parce que je pense que faire de la visio chez mes parents, ça aurait été très compliqué vue qu' ils ont une vie très active à la maison ils font de la musique et ils ne sont pas très soucieux du fait d'avoir des gens qui étudie donc du coup ça crée vite des difficultés dans l'apprentissage. La chambre est très petite aussi donc tu es vite enfermé dans un minuscule espace"
    b. **vous viviez seul ? chez vos parents ? en colocation ?** "Parent et coloc"
12. **Les cours en visioconférence ont-ils eu un impact sur votre motivation ?**
    a. **Si non ou oui , pourquoi ?** "Oui car j'aime beaucoup interagir pendant les cours et le fait de ne pas pouvoir ben je voulais laisser la place à me collègue mais qui ne posait pas forcément des questions et cela me démotivait à poser des questions."
13. **Avez-vous eu la possibilité d'étudier/ apprendre /travailler avec d'autres étudiants, enseignants ou autres** ? "Non"
14. **Avez-vous des personnes ressources/ privilégiées dans la classe pour vous aider en cas de besoins ?** " Il y avait passablement d'entraide sur tous les sujets donc oui.Toute la classe bonne resources et j'avais aussi quelques personnes dont j'étais plus proche qui pouvait m'aider, à revoir des choses que je n'avais pas comprise ou voir des choses d'un autre point de vue, c'était très aidant"
15. **Avez-vous gardé contact avec vos collègues de classe durant les cours à distance?**
    a. **Si oui, par quel moyen ?**oui, appel whatsapp, présentiel pour ceux qui travaillaient en ambulance, on se croisait de temps en temps.
    b. Numérique, présentiel ?
    c. **Cela été un soutien émotionnel pour vous ?**oui
    d. **Cela été un soutien d'apprentissage ?**non
    e. **Avez-vous pu le trouver vers quelqu'un d'autre ? "**non, j'ai essayé d'avoir une organisation de travail et de me pousser seul. "

Formation pratique (stage et pratique simulée à l'école)

16. **Avez-vous été affecté (confinement) sur le nombre d'heures de formation pratique ?** "Oui"
17. **Avez-vous l'impression que cela à eu un impact sur le développement de vos compétences pratiques (gestes techniques etc)?** "Oui "

a. **Oui-> Sentez-vous moins à l'aise en pratiquant des gestes techniques (voie veineuse, planchage…) ou bien sur l'évaluation primaire, anamnèse ?** "Plus sur le côté analytique car je pense ce qui nous amène beaucoup en stage c'est les questions analytiques de chaque spécialité, (pédiatrie, anesthésie) mais la pédiatrie, les urgences, ça reste des particularités sur les questions anamnestiques et du coup j'ai trouvé que j'avais un manquement de ses questions sur ses problèmes. J'ai du trouver un moyen alertatif pour rattarper mon retard par rapport à ce savoir que je n'ai pas eu"

18. **Avez-vous eu des stages annulés ?** "Oui"
    a. **Si oui combien de jours /semaines?** "1 mois d'urgence et 3 semaines en anesthésie, 1 semaine au 144: total 2 mois "
19. **Avez-vous travaillé à la place dans un service ?** "oui"
    a. **oui -> Pensez-vous que cela a été bénéfique pour votre apprentissage pratique ? "**Oui sauvez mon diplôme"
20. **Considérez-vous que l'annulation de stages ont eu un impact sur le développement de vos compétences professionnelles ?** "Non garce au travail en ambulance."
21. **Etiez-vous moins engagé dans la formation?** "Oui, par le fait de l'organisation scolaire compliquée, devenu des annulations, on ne savait pas si c'était en visio, on avait l'impression que c'était beaucoup de travail pour l'école de fournir les cours, la qualité des cours moins bonne de l'habitude, et du coup c'est vrai qu' entre ça et ce qu'on apprenait et ce qu'on voyait en travaillant c'était l'opposé . Il y avait un fossé entre l'école et la pratique. et j'avais l'impression d'apprendre des choses qui n'étaient pas nécessaires pour mon travail, j'avais l'impression de perdre mon temps à l'école alors que j'apprenais beaucoup en travaillant."
22. **Avez-vous eu l'envie d'arrêter la formation ?** "Non"
23. **Avez-vous senti une différence une fois sur un nouveau lieu de stage ?** "Non"
24. **Considérez-vous que le confinement, le covid 19 a eu un impact sur la valeur de votre diplôme (jeunes diplômés) ?** "Oui, moi j'ai beaucoup beaucoup de gens dans le milieu professionnel qui ont critiqué le fait de la gestion des examens et la passation de l'examen par l'ESAMB avait été médiocre et que beaucoup de personnes n'auraient pas dû passer parce qu'ils avaient pas les compétences et donc moi je me suis beaucoup remis en question, concernant mes compétences, parce que du côté professionnel tout le monde disait qu'on était moins compétent qu'avant et de l'autre côté l' école disait qu'on avait les mêmes compétences et que cela changeait rien alors qu'on a quand même eu des modules de cours annulés des stages annulés. Et je pense pour moi, j'ai été la seule rare personne à bosser pendant le confinement. Et que j'ai entendu beaucoup de critiques à l'encontre des mes camarades de classe,en disant que leur compétences étaient la baisse au niveau d'exigence demandé. Et je me sentais mal de ne pas être dans ce cas de figure là."
25. **Après cette période de pandémie, beaucoup de techniques ont été utilisées, selon vous y'a-t-il des techniques à garder qui ont favorisé votre apprentissage ?** "E-learning super, une autre chose les cours enregistrés. Les contacts téléphoniques avec les profs pour savoir comment on allait très chouette de se sentir écouter et nos besoins d'écouter."

Etudiant 4

Âge : 27 ans
Volée : 18-21

1. **Comment avez-vous vécu le confinement ?** “C’était long, donc je l’ai bien vécu mais c’était long et ennuyeux.”
2. **Êtes-vous senti stressé ?** “Pas plus que ça non “
    a. **Ça veut dire quoi pas plus que ça ?** “Que j’étais pas plus stressé que d’habitude “
    b. **Donc cela n'a pas eu d’impact sur ta vie privée ?** “Non, ce qui a eu plus d’impact sur ma vie privée c'est le fait d’être confiné, mais pas le stress”
3. **Avez-vous eu peur ?** “Je dirais un peu au début parce que tu sais pas ce que c’est cette maladie mais j’ai vite été rassuré avec l’évolution”
    a. **Oui-> Quelle était votre peur ?** “L’’inconnue, le fait de pas savoir , très clairement qui touche et comment elle touche cette maladie et comment elle fonctionne réellement. C’était vraiment au tout début que j’avais ces peurs et une fois qu’on avait plus d’explication dessus et puis que j’ai surtout pu être confronter à la maladie avec les tenues tyvek et les machins et tout que je me suis rendu compte que moi ça allait,bah ça a beaucoup diminué ma peur à moi.”
    b. **Comment avez-vous ressenti votre peur (physiquement, psychologiquement)?** “Par de l’inquiétude et de la méconnaissance”
    c. **Quel impact cela a eu sur votre manière d’apprendre ?** “Je pense pas, parce que j’ai beaucoup eu des cours en visioconférence avec les soins infirmiers, et du coup j’ai beaucoup eu l’ habitude de travailler sur mon ordi et de pouvoir prendre des notes en même temps que j’écoutais directement sur mon ordi. Donc moi au contraire, j’ai bien aimé cette manière de fonctionner parce que du coup ça me permettait de vraiment couper quand quelques choses m’intéressait pas et aller boire un verre d’eau, me lever, faire ma vie et reprendre le cours quand c’était quelques choses que j’avais pas compris ou que je ne connais pas très bien.”
    d. **Avez-vous fait quelque chose pour la diminuer ?** “Oui, lire les études, les derniers articles qui paraissaient. Mais je n’ai pas fait grand chose de plus. Et aller au travail et être confronté à la maladie. C’est les deux choses qui m’ont rassuré”
4. **Avez-vous eu de la joie ?** “Oui, il y a des moments mais le confinement en lui-même je dirais pas que c’était un moment de joie. C’est pas le premier sentiment que j’ai à l’esprit quand je pense au mot confinement”
    a. **Oui -> Comment s’est-elle manifestée ?** “Dans la vie de tous les jours, des moments rigolos avec Sarah ou avec des collègues par visioconférence”
5. **Êtes-vous senti anxieux ?** “Anxieux pas tellement non, au tout début oui ça va avec le sentiment de la peur. C’était de l’anxiété et de la crainte face à la maladie”
    a. **Oui->Comment s’est manifestée votre anxiété ?** “Face à la maladie”
    b. **Cette anxiété a-t-elle eu un impact dans votre vie privée ?** “Non”
    c. **Quel impact cela a eu sur votre manière d’apprendre ?** “Non”

d. **Avez-vous fait quelque chose pour la diminuer ?** “Pareil qu'avant donc lires des études et aller au contact de la maladie pour enlever cette crainte”

6. **Êtes-vous senti triste?** “Non”
7. **Êtes-vous en colère?** “Non plus ”
8. **Êtes-vous senti seul (dimension soutien social) ?** “Bah ça diminue, tu te sens un plus seul forcément, je me suis senti plus seul en confinement que quand tu es à l’école et que tu peux aller partager tous les bons moments avec tes collègues mais moi c’est bien tombé, c’était pil le moment où j’ai emménagé avec Sarah donc tout une nouveauté avec du faite de vivre à deux. Et du coup ça un peu compenser, je dirais “
   a. **Oui ->Vous viviez seul ?** “Non”
   b. **Avez -vous un réseau d'amis important ?** “Oui”
   c. **Sur combien d'amis fidèles pouvez-vous compter (au sens de l’ami.e que vous pourriez réveiller au milieu de la nuit) ?**”10”
   d. **Votre famille se compose-t-elle de personnes ressources pouvant vous aider à apprendre ?** “Non”
9. **Comment se passaient les cours à distance (théoriques) ?** “Moi vu que j’avais déjà eu une expérience tout autre avec les soins infirmiers et que j’avais déjà travaillé comme ça par ordinateur en re-visionnant des cours qui avaient été enregistré au près à lable, bah du coup pour moi cela a bien été.”
10. **Qu’est-ce qui vous manquaient le plus en ayant les cours en visioconférence ?** “Le contact social”
11. **Aviez-vous un environnement propice à l’apprentissage (au domicile) ?** “Oui”
   a. **Si non ou oui , pourquoi ?** “Pour plusieurs raisons,parce que j’avais un endroit où je peux me mettre tranquille et y a pas de bruit et rien qui va me déranger, et je suis bien installé”
   b. **Vous viviez seul ? Chez vos parents ? En colocation ?** “Sarah”
12. **Les cours en visioconférence ont-ils eu un impact sur votre motivation ?** “Oui quand même, c’est beaucoup moins motivant, tout seul à relire un cours Si je prends soins infirmiers, c’était quand je ne pouvais pas aller en cours, je pouvais les relires par la suite de mon côté mais c’était un cours ou deux mais quand c’est toute une semaine entière sur des cours en visio moi ça me démotive beaucoup.
13. **Avez-vous eu la possibilité d’étudier/ apprendre /travailler avec d’autres étudiants, enseignants ou autres ?** “Non”
14. **Avez-vous des personnes ressources/ privilégiées dans la classe pour vous aider en cas de besoins ? “**Oui on a des groupes whatsapp avec deux trois personnes de la classe avec qui j’entendais bien et puis en gros quand quelqu’un ne comprenait pas ou bien que je ne comprenais pas, les questions se posait pas le groupe whatsapp. C’est vrai que moi personnellement quand on était en cours et que je ne comprenais pas souvent tu avais l’occasion de poser la question à voix-basse à ton collègue et puis ils te répondaient en deux mots et cela te permettait d’accrocher avec le reste du cours. Tandis que là, en visioconférence tu as plus cette possibilité là. Donc c’était le truc qui moi m’a dérangé le plus de faire tous les cours en visio.La solution s’était What’app,où on posait la question sur le groupe de la classe et celui qui avait compris donnait vite fait une réponse et pis si on

voyait que trop de personne n'avait pas compris, on interrompt le cours pour poser la question."

15. **Avez-vous gardé contact avec vos collègues de classe durant les cours à distance?**
    a. **Si oui, par quel moyen** "Oui par skype"
    b. Numérique, présentiel ?
    c. **Cela été un soutien émotionnel pour vous ?** "Oui "
    d. **Cela été un soutien d'apprentissage ?** "Oui à la fin du cours, on commençait déjà si y avait des gens qui avait pas compris, on s' expliquait vite fait et des fois l'entendre le ré-expliquer par une autre personne s'est souvent plus facile à comprendre et cela de bien intégrer ce que tu as entendu."
    e. **Avez-vous pu le trouver vers quelqu'un d'autre ?** "Sarah"

Formation pratique (stage et pratique simulée à l'école)

16. **Avez-vous été affecté (confinement) sur le nombre d'heures de formation pratique ?** "Oui,moi je trouve que pour la pédiatrie on a loupé toute la pratique, et je trouve que , enfin personnellement la pédiatrie reste un stress que je n'ai pas dans les autres branches (médicale ou trauma). J'ai que la pédiatrie que j'ai loupé,moi j'ai le sentiment que ça vient de là,même si la pédiatrie reste quelque chose de très stressant pour tout le monde, même ceux qui ont fait la pratique."
17. **Avez-vous l'impression que cela à eu un impact sur le développement de vos compétences pratiques (gestes techniques etc)? "**Oui typiquement la pédiatrie, nébuliser un enfant et tout ça enfin moi, personnellement, je n'ai jamais nébulisé d'enfants, en pratique ou en vrai"
    a. **Oui-> Vous sentiez-vous moins à l'aise en pratiquant des gestes techniques (voie veineuse, planchage…) ou bien sur l'évaluation primaire, anamnèse ?** "Je me sens moins à l'aise sur le deux" **Encore maintenant ?** "Maintenant ça va gentillement mais j'ai pas encore fait de grosses situations instables, on verra le jour où je serai confronté à une vrai cours ped instable, je pense que je ne serai pas aussi à l'aise que ce que je peux l'être dans le médicale par exemple."
18. **Avez-vous eu des stages annulés ? Si oui combien de jours /semaines?** "Oui, 3 semaines d'anesthésie, au sis 1 mois au lieu de deux mois."
19. **Avez-vous travaillé à la place dans un service ?** "Oui chez SAG"
    a. **Oui -> Pensez-vous que cela a été bénéfique pour votre apprentissage pratique ?** "Oui clairement, pour tout ce qui est resp, parce qu'à ce moment là, j'ai fait quasiment que ça."
20. **Considérez-vous que l'annulation de stages ont eu un impact sur le développement de vos compétences professionnelles ? Si oui de quelle manière ?** " Je pense que si j'avais pu avoir mon stage en anesth, je serai encore plus à l'aise que je ne suis maintenant pour ventiler et avec les drogues.
21. **Comment avez-vous compensé ce manque ?** "Oui, au SIS j'ai pu faire une journée en Anesthésie avec les médecins répondants donc j'ai pu voir tout ce qui était drogue et ventilation."
22. **Etiez-vous moins engagé dans la formation ?** "Non comme je travaillais, c'était pas des moments où je n'ai rien fait comme certains de ma classe n'ont

pas pu travailler. Moi vu que je travaillais chez SAG, je me sentais toujours investi.” **Avez-vous eu l’envie d’arrêter la formation ?** “Non “

23. **Avez-vous senti une différence une fois sur un nouveau lieu de stage ?** “Non “
24. **Considérez-vous que le confinement, le covid 19 a eu un impact sur la valeur de votre diplôme (jeunes diplômés) ?** “Oui, typiquement la pédiatrie, tout ce qu’on a manqué, j’ai le sentiment que c’est des trucs qu’on doit rattraper nous sur le côté.”
25. **Après cette période de pandémie, beaucoup de techniques ont été utilisées, selon vous y'a-t-il des techniques à garder qui ont favorisé votre apprentissage ?** “Les cours enregistré que tu peux réécouter”

Etudiant 5

Âge : 24 ans
Volée : 17-20

1. **Comment avez-vous vécu le confinement ?** “Moi pour être franc,je l’ai bien vécu, c’était pas un problème vraiment, on a eu confinement light et modéré en suisse, moi j’étais en stage et du coup j’allais travailler et c’était pas…enfin je n’ai pas vécu ça comme un vrai confinement où j’étais contraint dans ma liberté. Je n’ai pas vécu comme une grosse différence mise à part l’aspect avec les potes mais sinon je l’ai bien vécu”
2. **Êtes-vous senti stressé ?** “Non je pense pas, je pense que si j’étais stresser c'était pour des facteurs qui était pour lequel j’aurais été stressé à cette période-là, c’était la période où j’allais finir mes étude, et puis il y avait quelques enjeux sur le plan des études: le travail de diplôme, des stages à valider et tout ça…mais des facteurs qui auraient été présent même sans confinement”
    a. **Oui -> Comment avez-vous ressenti ce stress (physiquement, psychologiquement) ?** “Je ressentais pas de stress en lien avec la pandémie”
3. **Avez-vous eu peur ? “**Non, je pense j’ai été…je pense peur c’est un trop grand mot, j’ai eu des appréhensions qui était lié en fait et puis la pandémie moi je l’ai vécu différemment du grand public vue que j’étais sur le terrain, et puis ces espèces de normes d’hygiène qui apparaissaient qu’on avait jamais vraiment parlé mais j’ai pas eu peur plus que ça, juste une appréhension lié à l’incertitude”
    a. **Oui-> Quelle était votre peur ?** “L’appréhension lié à l’incertitude “
    b. **Comment avez-vous ressenti votre peur (physiquement, psychologiquement)?** “Ouais, je pense c’était plus sur l’aspect comment je me projette dans l’avenir, sur le fait de me dire ben un aspect très individuel, dans le sens où est ce que je vais devoir rester en suisse, est ce que je vais pouvoir aller en vacance. Aussi des aspects professionnels , est ce que je vais garder le masque. Au début, enfin j’ai jamais eu de stress physique, d’un stress dont j’étais conscient, plus de quelque chose d’individuel.”
    c. **Quel impact cela a eu sur votre manière d’apprendre ?** “Je pense que j’avais plus grand chose à apprendre à ce moment-là, dans le sens où, pas de l'arrogance, mais on avait fait quasi tous nos cours, il reste deux -trois cours, des choses à apprendre en visio, donc je ne pense pas que ça a eu un gros impact là dessus.
    d. **Avez-vous fait quelque chose pour la diminuer ?** “Non , je me suis renseigné sur ce que c’était le covid et puis sur comment, ce qui se passait. Je pense que c'était le meilleur moyen de combattre la peur de l’inconnu.”
4. **Avez-vous eu de la joie ?** “oui par moment, aspect de te dire que c’était presque un peu cool, des espèces de vacances, qui te sont un peu offerte “
    a. **Oui -> Comment s’est-elle manifestée ?** “Aspect cool vacances offerte, plus de joie que de peur.”
5. **Êtes-vous senti anxieux ?** “Non”
6. **Êtes-vous senti triste?** “Non je crois pas “

7. **Êtes-vous en colère?** “Oui, j’ai eu des moments de colère, genre plus lié à des incohérences sur des positions politiques, je pense. De me dire de on enlève le masque, c’est des soirées à dix à l'intérieur, si on a le pas. tu vois là encore, il n’y a plus de confinement mais je trouve incohérent de garder le masque dans les transports publics mais pas ailleurs. Du coup, je pense que oui j’ai pu être un peu soulé quoi!”
    a. **Oui -> Comment s’est-elle manifestée ?** “C’était des débat avec des potes, genre de ce dire que c’était ridicule mais c’était pas physiquement de colère”
    b. **Cette colère a-t-elle eu un impact dans votre vie privée ?** “Non, je pense pas franchement”
    c. **Quel impact cela a eu sur votre manière d’apprendre ?** “Non je pense pas non plus”
    d. **Avez-vous fait quelque chose pour la diminuer ?** “Je crois pas non, mais d’avoir discussion ça a aidé”
8. **Êtes-vous senti seul (dimension soutien social) ?** “Oui, ça oui, même maintenant j’ai l’impression d’avoir, un grand mot, mais des séquelles, dans le sens où socialement, je trouve que, genre un truc bête mais avant j’allais en boite,et puis maintenant des fois j’ai même plus envie, je ne suis même pas motivé, ça a lancé une dynamique une peu, une ambiance où tu es tranquille et tu moins envie de voir du monde, tu t’es habitué à ça, alors qu’avant j’avais pas forcément envie, et j’allais quand même et c’était cool. Alors que maintenant,il y a une dynamique où tu as moins envie d’être en grand groupe.”
    a. **Oui ->Vous viviez seul ?** “Non à ce moment je vivais chez mes parents et chez mon ex “
    b. **Avez -vous un réseau d'amis important ?** “Oui”
    c. **Sur combien d'amis fidèles pouvez-vous compter (au sens de l’ami.e que vous pourriez réveiller au milieu de la nuit) ?** “Sept”
    d. **Votre famille se compose-t-elle de personnes ressources pouvant vous aider à apprendre ?** “Oui “
        i. **Si oui combien ? “** Trois”
        ii. **Frère / sœur ?** “Frère”
        iii. **Père / mère ?** “Père et mère”
        iv. **Quel est leur niveau de formation ?** “ Ma mère a un CFC, mon père a une maîtrise fédérale et mon frangin, il est en master”
9. **Comment se passaient les cours à distance (théoriques) ?** “C’est compliqué, je trouve, de garder une certaine motivation et d’être attentif. Tu es vite, tu as vite ton attention qui divague et tout ça.”
10. **Qu’est-ce qui vous manquaient le plus en ayant les cours en visioconférence ?** “La présence, je pense d’avoir quelqu’un, c’est une présence humaine”
11. **Aviez-vous un environnement propice à l’apprentissage (au domicile) ?**
    a. **Si non ou oui , pourquoi ? “**Oui, je pense quand même,j’ai une chambre où je suis déjà seul, j’ai un bureau, je peux m'asseoir et travailler. Et je peux avoir un environnement calme, si je le demande.”
    b. **Vous viviez seul ? Chez vos parents ? En colocation ?** “Chez mes parents”

12. **Les cours en visioconférence ont-ils eu un impact sur votre motivation ?**
    a. **Si non ou oui , pourquoi ?** “Oui, un impact négatif, dans le sens où, c’est pas très motivant de se lever de son lit pour se mettre sur le bureau et d’écouter quelqu’un avec lequel tu n’as pas d’interaction même si il se donnait de la peine, l’école se donne la peine. Il essayait de faire de leur mieux”
13. **Avez-vous eu la possibilité d’étudier/ apprendre /travailler avec d’autres étudiants, enseignants ou autres ?** “Oui avec mon ex”
14. **Avez-vous des personnes ressources/ privilégiées dans la classe pour vous aider en cas de besoins ? “**Oui”
15. **Avez-vous gardé contact avec vos collègues de classe durant les cours à distance?**
    a. **Si oui, par quel moyen ?** “Oui”
    b. **Numérique, présentiel ?** “Les deux “
    c. **Cela été un soutien émotionnel pour vous ?** “Ouais quand même, je pense”
    d. **Cela été un soutien d’apprentissage ?** “Oui, je pense j’ai plus été moi-même le soutien pour les autres”
    e. **Avez-vous pu le trouver vers quelqu’un d'autre ?** “Non, mais on était au bout de nos études “

Formation pratique (stage et pratique simulée à l’école)

16. **Avez-vous été affecté (confinement) sur le nombre d’heures de formation pratique?** “Oui”
17. **Avez-vous l’impression que cela à eu un impact sur le développement de vos compétences pratiques (gestes techniques etc)?** “Oui, je n’ai pas fait de stage en pédiatrie”
    a. **Oui-> Vous sentiez-vous moins à l’aise en pratiquant des gestes techniques (voie veineuse, planchage…) ou bien sur l’évaluation primaire, anamnèse ?** “Je pense qu'en l'occurrence c’est plus des gestes techniques, par exemple poser un cath sur un enfant, chose que je n’ai jamais fait même maintenant.”
18. **Avez-vous eu des stages annulés ? Si oui combien de jours /semaines?** “Oui, un annulé le stage de pédiatrie (3 semaines) et un écourté qui était mon dernier stage en ambulance, il me manquait deux semaines à faire”
19. **Avez-vous travaillé à la place dans un service ? “**Oui”
    a. **Oui -> Pensez-vous que cela a été bénéfique pour votre apprentissage pratique ?** “Oui, je pense, ça fait l’expérience. Je pense que c’était kif-kif à ce stade de la formation”
20. **Considérez-vous que l’annulation de stages ont eu un impact sur le développement de vos compétences professionnelles ? Si oui de quelle manière ?** “Oui, sur compétence technique en l'occurrence lié à de la pédiatrie, lié à de la gestion de l’entourage sur la ped genre : un parent avec son enfant. **Comment avez-vous compensé ce manque ?** “Je l’ai pas compensé”
21. **Etiez-vous moins engagé dans la formation?** “Non” **Avez-vous eu l’envie d’arrêter la formation ?** “Non pas du tout.”
22. **Avez-vous senti une différence une fois sur un nouveau lieu de stage ?** “Je ne suis plus retourner en stage, pas eu l’occasion.”

23. **Considérez-vous que le confinement, le covid 19 a eu un impact sur la valeur de votre diplôme (jeunes diplômés) ?** “Je ne pense pas, je pense que certain pourrait dire que oui mais moi je pense pas. Mon diplôme j’allais le réussir avec ou sans pandémie”
24. **Après cette période de pandémie, beaucoup de techniques ont été utilisées, selon vous y'a-t-il des techniques à garder qui ont favorisé votre apprentissage ? “**E-learning c’était bien”

Etudiante 6

Âge : 24
Volée : 2016-2020

1. **Comment avez-vous vécu le confinement ?** ”Si c’était uniquement par rapport à l’école en soi ça m’a fait un peu chier car il y a quand même pas mal de chose qu’on n’a pas pu voir, typiquement, c’était un peu la période catastrophe, les grossesses avec la complication, je trouve pour tout ce qui est evénement majeur, c’est quelques choses que tu dois pratiquer en vrai et nous on a tout fait par ordinateur et par visioconférence alors certes, ils se sont une chier débrouiller pour trouver des solutions pour qu’on comprenne tous ces principes et tout ça. Mais je trouve que ça ne remplace pas le vrai qu’on aurait dû vivre à cette période. Encore par rapport à l’école, on a des stages annulés, moi j’ai loupé mon stage en psychiatrie et aussi obstétrique et c’est le stage que j’attendais le plus dans la formation,et j’ai jamais eu le stage en obstétrique et ça j’ai eu les boules et pour Valtra j’ai eu les méga boules. Donc globalement, je trouve qu’au niveau scolaire c’était très compliqué parce que tu n’as pas le contexte réel. Chez toi, tu as tellement de distraction et c’est hyper dur de se concentrer sur ce qu'on fait. Après voilà, l’ école a fait tout son possible pour qu’on aie toutes les notions qu’on avait besoin d’avoir et ça c’est méga chouette. Après au niveau personnel, c’était quand même cool, j’ai pû être utile et soutenir les services ambulances et quand on a été en confinement on a pu bosser une chier, et ça c’est cool. Cela a apporté un côté pratique et de très terrain avant de finir les cours. On était du coup directement dans les prises en charge Covid et finalement on a commencé à travailler en période covid et quand on a été diplômé cela n’a rien changé.”
2. **Êtes-vous senti stressé ?** “Oui mais parce qu’on ne savait pas exactement où est ce qu’on allait. On ne savait pas au niveau des cours, c’était un peu bancal. Est ce qu’on allait partir en stage pas en stage ? Finalement, qu’est ce qu’on va faire si on est confiné et tout ça. Et oui forcément ça engendrait du stress car on ne savait pas où on allait aller.”
    a. **Oui->Comment avez-vous ressenti ce stress (physiquement, psychologiquement)?** "Ni l'un ni l’autre car finalement j’étais contente je bossais mais je pense plus psychologique que physique.”
    b. **Avez-vous eu une perte de plaisir ?** “Non”
    c. **Avez-vous eu une perte d'intérêt ?** “Non”
    d. **Ce stress a-t-il eu un impact dans votre vie privée ?** “Je ne crois pas”
    e. **Quel impact cela a eu sur votre manière d’apprendre ?** “Bah ça oui, clairement, c’était un apprentissage différent c’était pas par la pratique uniquement par la visioconférence et de la théorie.Et pour quelqu’un comme moi qui a besoin de pratiquer pour comprendre, c’est sûr que ça a tout chambouler”
    f. **Qu'est-ce que vous avez fait pour être moins stressé ?** "Travailler, bosser ça permettait d’évacuer, de penser à autre chose et faire ton job et d’avancer.”
3. **Avez-vous eu peur ?** “Non”

4. **Êtes-vous senti anxieux ?** “Non, je ne suis pas non plus quelqu'un de stressé, ça m'a plus épuisé que stressé.”
5. **Êtes-vous senti triste?** “Non”
6. **Êtes-vous en colère?** “Non “
7. **Êtes-vous senti seul (dimension soutien social) ?** “Non pas vraiment car j’allais au travail, j’avais mes collègues,et finalement ça changeait pas plus que ça. Après oui, de pas voir les camarades de classes c’était une assez drôle de période, on a fait que de la visio, c’était un peu long. Tu as envie de voir tes collègues, tes camarades de classe. Oui ça c’était difficile”.
    a. **Oui ->Vous viviez seul ?** “Chez mes parents”
    b. **Avez -vous un réseau d'amis important ?** “Non, je dirais pas important”
    c. **Sur combien d'amis fidèles pouvez-vous compter (au sens de l’ami.e que vous pourriez réveiller au milieu de la nuit) ?** “4”
    d. **Votre famille se compose-t-elle de personnes ressources pouvant vous aider à apprendre ?** "Négatif”
8. **Comment se passaient les cours à distance (théoriques) ?** “Mal, parce que de bases j’ai de la peine avec tout ce qui est de théorie,mais en plus par visio, tu peux pas poser toutes les questions que tu as envie de poser et finalement tu as que cet apport théorique que j’avais de la peine de base “
9. **Qu’est-ce qui vous manquaient le plus en ayant les cours en visioconférence ?** “La pratique,ah au niveau de la théorie. Il me manquait le plus c’est l’échange, c’est quand même différent du face à face, l’échange avec les camarades où tu peux poser des questions, poser des questions au prof. C’est pas wouah”
10. **Aviez-vous un environnement propice à l’apprentissage (au domicile) ?**
    a. **Si non ou oui , pourquoi ?** “Est ce que c’est propice à l’apprentissage ? Du moment que tu es à la maison ce n’est pas propice à l’apprentissage, tu as pleins de choses juste pour divertir l’apprentissage. Donc oui,j’avais un environnement calme mais malgré ça j’avais pleins de distractions “
    b. **Vous viviez seul ? Chez vos parents ? En colocation ? “**Chez mes parents”
11. **Les cours en visioconférence ont-ils eu un impact sur votre motivation ?** “Non”
12. **Avez-vous eu la possibilité d’étudier/ apprendre /travailler avec d’autres étudiants, enseignants ou autres ?** “Oui avec d’autres étudiants”
13. **Avez-vous des personnes ressources/ privilégiées dans la classe pour vous aider en cas de besoins ?** “Oui “
14. **Avez-vous gardé contact avec vos collègues de classe durant les cours à distance?**
    a. **Si oui, par quel moyen ?** “Oui”
    b. **Numérique, présentiel ?** “Plutôt par whatsapp, message et tout ça. Et après quelques uns en présentiel comme Jo, Quentin."
    c. **Cela été un soutien émotionnel pour vous ?** “Clairement, parce que tu voyais que tu étais pas la seul à pas comprendre certaine chose et du coup c’est vrai que celui qui comprenait expliquait à celui qui ne comprenait pas et vice versa. Heureusement qu’on avait ce truc what’s app car sinon ça aurait compliqué les choses”
    d. **Cela été un soutien d’apprentissage ? “**Oui”
